# Impact of multifaceted clinical decision support and education on antibiotic duration in outpatients with respiratory tract infections in Saudi Arabia: a prospective pre- and postimplementation study

**DOI:** 10.1017/ash.2025.10219

**Published:** 2025-11-17

**Authors:** Ahlam Alghamdi, Mohammed Alraey, Mohammad Aatif Khan, Lina I. Alnajjar, Reem Binsuwaidan, Maram Almutairi, Faridah Alsafi, Nouf Alhumaidah, Hala Alqhatani, Eman Alghamdi, Wejdan Alassaf, Abdulaziz Saad Aleissa

**Affiliations:** 1 Department of Pharmacy Practice, College of Pharmacy, Princess Nourah bint Abdulrahman University, Riyadh, Saudi Arabia; 2 Department of Infectious Diseases, King Abdullah bin Abdulaziz University Hospital, Riyadh, Saudi Arabia; 3 Microbiology Laboratory, Department of Pathology and Laboratory Medicine, King Abdullah Bin Abdul-Aziz University Hospital, Princess Nourah bint Abdulrahman University, Riyadh, Saudi Arabia; 4 Department of Pharmaceutical Sciences, College of Pharmacy, Princess Nourah bint Abdulrahman University, Riyadh, Saudi Arabia; 5 Pharmaceutical Care Services, King Abdullah bin Abdulaziz University Hospital, Riyadh, Saudi Arabia; 6 PharmD Candidate, College of Pharmacy, Princess Nourah bint Abdulrahman University, Riyadh, Saudi Arabia; 7 Saudi Food and Drug Authority, Riyadh, Saudi Arabia; 8 Emergency Department, King Khalid University Hospital, King saud university Medical city, Riyadh, Saudi Arabia; 9 Family Medicine department, King Abdullah bin Abdulaziz University Hospital, Riyadh, Saudi Arabia

## Abstract

**Objectives:**

Prolonged antibiotic therapy is associated with an increased risk of antimicrobial resistance and adverse events. We evaluated the implementation of a multifaceted antimicrobial stewardship (ASP) initiative aimed at reducing antibiotic duration for respiratory tract infections (RTIs) in outpatient settings.

**Designs:**

Quasi-experimental study.

**Settings:**

Academic medical center in Riyadh, Saudi Arabia.

**Patients:**

Patient with RTIs in outpatient settings

**Methods:**

A multifaceted ASP intervention—including a clinical decision support tool and an educational session—was implemented to guide physicians in prescribing the shortest effective duration of oral antibiotics. We compared antibiotic utilization and adherence to evidence-based duration prescribing in a preintervention phase (June 2021–December 2021) and a postintervention phase (January 2022–June 2022) .

**Results:**

We included 2320 patients in our study, of which 1359 were in the preintervention period and 961 in the postintervention period. Following implementation of the multifaceted stewardship initiative, the days of therapy (DOT) per 1,000 outpatient visits decreased from 131 to 50 and the mean duration of antibiotic therapy declined from 6.4 to 6.0 days (*p* < 0.001). Adherence to the recommended duration improved, with the percentage of prescriptions meeting recommended duration increasing from 49.7% to 56.3% (*p* = 0.002).

**Discussion:**

The multifaceted ASP initiative can reduce unnecessary antibiotic exposure and improve the adherence to the recommended duration.

## Introduction

Antimicrobial resistance (AMR) is a critical global health challenge, with international and national efforts actively promoting appropriate antibiotic use to reduce the emergence and spread of resistance.^1–4^ In Saudi Arabia, the national AMR Action Plan—guided by the Global AMR Action Plan—promotes the development of antimicrobial stewardship program (ASP), automation, and surveillance.^2,5,6^

One of the major contributing factors to the emergence and spread of AMR is the prolonged use of antibiotics.^7^ Multiple randomized trials comparing the short versus long duration of oral antibiotic therapy demonstrated that a shorter duration of antibiotics are as effective as longer duration of antibiotics with fewer adverse events.^7,8^

Although ASPs have been widely implemented in Saudi Arabia to ensure antibiotics are prescribed appropriately and reduce the emergence of AMR, there is a notable gap in initiatives focused on optimizing the duration of antimicrobial therapy.^9^ Therefore, our institutional ASP team sought to assess the impact of multifaceted initiatives including clinical decision support (CDS) and education on antibiotic duration, with a focus on antibiotic utilization and adherence for respiratory tract infections (RTIs) in outpatient settings.

## Method

We conducted a quasi-experimental study at an academic medical center with 400 beds in Riyadh, Saudi Arabia. We compared oral antibiotic prescribing using evidence-based duration defaults for RTIs in a preintervention phase (June 2021–December 2021) and a postintervention phase (January 2022–June 2022) without incorporating a washout period in outpatient settings. The implemented antibiotic duration in the EHR, as approved by the institutional ASP committee, includes pediatrics and adults, is shown in Table 1. An RTI order set was implemented in the EHR’s Preferred Orders menu to standardize prescribing. Selecting the RTI diagnosis triggered a list of recommended antibiotics with approved treatment durations aligned with institutional guidelines. Clinicians chose the agent, with the option to modify dose or duration as clinically indicated.

**Table 1.** Institutional antimicrobial stewardship guidelines on recommended duration of oral antibiotic therapy for respiratory infections in outpatient settings

Infection	Recommended duration for adult, days	Recommended duration for pediatric, days
Upper respiratory tract infection		
Pharyngitis	5	5
Otitis media	7	<2y: 10 >2y: 7
Sinusitis	5	5
Community-acquired pneumonia	5	5

Strep throat infection duration includes penicillin and other antibiotics.

From the outpatient data set, we extracted all antibiotic prescriptions and analyzed those associated with community-acquired pneumonia (CAP) or upper respiratory tract infection (URTI) and encountered diagnoses in adults and children. We included outpatients on oral antibiotics for CAP or URTI. We excluded patients who received antibiotics for less than 72 hours, those with recurrent sinusitis, complicated acute bacterial sinusitis, other nonrespiratory infections, and immunocompromised individuals.

We assessed the impact of our stewardship initiative through measuring antibiotic utilization and percentage of prescriptions adhering to embedded duration for included patients in outpatient settings. We used days of therapy (DOT) metric to evaluate the antibiotic utilization and percentage of prescriptions adhering to recommended duration to assess compliance.

Calculation Method for Antibiotic Consumption:

1. Formula: DOT per 1 000 outpatient visits = (Total antibiotic days for RTI/Total outpatient visits) × 1 000

### Duration-based antibiotic stewardship intervention

#### Automation

We implemented the institutional ASP-approved CDS in the EHR for RIT on January 1, 2022. This initiative aims to voluntarily guide physicians to choose the shortest effective duration of antibiotic for URTI and CAP. This was only implemented in the outpatient clinics and emergency department.

#### Education

An infectious diseases pharmacist conducted an educational session on the “Shorter is Better” initiative during a departmental meeting for outpatient clinics and emergency department. This session included physicians, nurses, and healthcare staff, ensuring comprehensive awareness and adherence to evidence-based antibiotic prescribing practices.

#### Statistical analysis

We analyzed categorical data using χ^2^ test and continuous data using the Student *t* test. We also utilized a multivariate logistic regression model to identify independent factors associated with the adherence to the recommended antibiotic duration. Odds ratios (ORs) and 95% confidence intervals (CI) were calculated for all associations in the logistic regression. All statistical analyses were performed using SPSS version 27.0 (IBM, USA).

## Result

During the study period, a total of 2 335 patients received oral antibiotics for respiratory infections in the outpatient settings. Of these, 15 were excluded based on the exclusion criteria. We included 2 320 patients in our study, of which 1 359 were in the preintervention period and 961 in the postintervention period (Table 2).

**Table 2.** Characteristics of outpatients with respiratory tract infections in the pre- and postintervention periods of the multifaceted stewardship initiative

Variable	Preintervention (*N* = 1 359)	Postintervention (*N* = 961)	*P*-value
**Age, years, median, IQR**	24 [8–39]	20 [6–38]	.004
<14 year	443 (32.6)	422 (43.9)	< .001
>14 year	916 (67.4)	539 (56.1)	< .001
Male, n (%)	577 (42.5)	424 (44.1)	.43
Obesity (BMI>35)	7 (0.5)	31 (3.2)	< .001
Emergency department	984 (72.4)	776 (80.7)	< .001
Outpatient	375 (27.6)	185 (19.3)	< .001
**Comorbidities**			
Asthma/COPD	62 (4.6)	182 (18.9)	< .001
Diabetes mellitus	51 (3.8)	69 (7.2)	< .001
Hypertension	46 (3.4)	59 (6.1)	.002
Hypo/hyperthyroidism	22 (1.6)	33 (3.4)	.005
Cardiovascular disease	7 (0.5)	6 (0.6)	.73
Chronic kidney disease	1 (0.1)	2 (0.2)	.37
**Respiratory tract infections**			
Upper respiratory tract infection	1 237 (91)	880 (91.6)	.65
Community-acquired pneumonia	122 (9)	81 (8.4)	.65
Prescribed antibiotic^*^			
β-lactam	904 (66.5)	623 (64.8)	.40
Macrolide	443 (32.6)	318 (33.1)	.80
Fluoroquinolone	9 (0.7)	16 (1.7)	.02
Doxycycline	3 (0.2)	4 (0.4)	.02

* Some patients received combination therapy.

The median age of participants was 23 years (IQR, 7–39), and 996 (43%) were male. Most patients had no significant underlying medical conditions, and 2,117 (91.5%) were diagnosed as having URTIs. Regarding antibiotic use, β-lactams were the most frequently prescribed agents (1,527; 65.8%), followed by macrolides (761; 32.8%), fluoroquinolones (25; 1.1%), and doxycycline (7; 0.3%) (Table 2).

Following implementation of the multifaceted stewardship initiative, significant improvements were observed in antibiotic prescribing metrics for RTIs. The DOT per 1,000 outpatient visits decreased from 131 to 50, indicating an overall reduction in antibiotic exposure. Similarly, the mean duration of antibiotic therapy declined from 6.3 to 6.0 days (*P* < 0.001). Adherence to the recommended duration also improved, with the percentage of prescriptions meeting recommended duration increasing from 54.2% to 58.8% (*P* = 0.02) (Table 3).

**Table 3.** Comparison of antibiotic prescribing metrics pre- and postintervention periods of the multifaceted stewardship initiative

Outcome	PreIntervention	Postmultifaceted intervention	*P*-value
DOT per 1,000 visits	131	50	NA
Mean duration, SD	6.3 (2.5)	6 (2.2)	.001
Prescriptions adhering (%)	737 (54.2)	565 (58.8)	.029

In multivariate logistic regression analysis, the intervention was associated with significantly higher odds of adherence to recommended antibiotic duration (OR = 1.2, 95% CI 1.10–1.41, *p* = 0.036). Adults had higher odds of adherence than pediatric patients (OR = 1.01, 95% CI 1–1.01, *P* = 0.03). Patients who visited outpatient clinics had higher odds of adherence compared to those seen in the emergency department (OR = 1.13, 95% CI: 1.13–1.69, *p* = 0.001). Gender (*P* = .10) was not significantly associated with adherence (Table 4).

**Table 4.** Multivariate logistic regression identifying predictors of adherence to recommend duration

Variables	OR	95% CI	*P* value
Period (pre vs post)	1.2	1.01–1.41	.036
Age group	1.01	1–1.01	.03
Gender	0.87	0.73–1.02	.10
Location	1.13	1.13–1.69	.001

Reference categories: period = preintervention; age group = pediatrics; gender = male; location = emergency department.

## Discussion

To our knowledge, this is the first study to evaluate the impact of multifaceted ASP initiative targeting antibiotic duration in the region. Our intervention, including CDS and education, was associated with improvements in key ASP metrics such as antibiotic utilization, mean antibiotic duration, and percentage of adherence to recommended duration.

Various stewardship programs have been implemented internationally to reduce unnecessary antibiotic exposure and improve patient’s outcomes.^10,11^ Some of these initiatives include clickable duration option in the EHR and automatic stop dates in the EHR.^10,11^ In our study, we used a multifaceted intervention to guide the physicians to choose the shortest effective duration, which led to a 38.2% reduction in the DOT per 1 000 visits and a 11.7% improvement in adherence to evidence-based duration. Although the mean duration of therapy decreased significantly from 6.4 to 6.0 days, this reduction is unlikely to be clinically meaningful in practice. However, this finding suggests that outpatient antibiotic prescribing practices may not alarming. Importantly, sustained implementation of ASP interventions remains essential, as even modest improvements can reinforce appropriate prescribing behaviors and contribute cumulatively to reducing unnecessary antibiotic exposure over time.

Similar outcomes in ASP metrics such as DOT reduction have been reported in other studies that implemented duration-based interventions.^11–14^

In our study, the shift in antibiotic use from β-lactams to other agents in the postintervention period may reflect evolving prescribing behaviors within the emergency medicine service or emerging resistance patterns, particularly during the COVID-19 pandemic.^15^

These kind of ASP efforts align with global and national initiatives to reduce AMR by minimizing unnecessary antibiotic exposure and are consistent with the Saudi Vision 2030 Health Sector Transformation Program, which prioritizes combating AMR through targeted ASPs, automation, and surveillance.^5,6,9^

These findings collectively highlight the value of multifaceted stewardship approaches, including education and automation.

Our study has several limitations. First, as a retrospective analysis of a quality improvement project, the results may be subject to residual confounding. Second, this was a single-center study, which may limit the generalizability of the findings to other hospitals in the region. Finally, because accurate diagnostic coding for RTIs was not available, we calculated DOT per 1,000 outpatient visits, which may not precisely reflect prescribing appropriateness for RTI cases.

## Conclusion

In summary, we implemented a multifaceted ASP initiative—including CDS and education—to guide physicians in selecting the shortest effective antibiotic duration for RTIs in outpatient settings. This intervention can reduce unnecessary antibiotic exposure and improve the adherence to the recommended duration. This is one of the few studies of such initiatives from the Eastern Mediterranean region, which is a site of significant rise of AMR.
